# Identification of novel mutations causing pediatric cataract in Bhutan, Cambodia, and Sri Lanka

**DOI:** 10.1002/mgg3.406

**Published:** 2018-05-16

**Authors:** Shari Javadiyan, Sionne E. M. Lucas, Dechen Wangmo, Meng Ngy, Kapila Edussuriya, Jamie E. Craig, Adam Rudkin, Robert Casson, Dinesh Selva, Shiwani Sharma, Karen M. Lower, James Meucke, Kathryn P. Burdon

**Affiliations:** ^1^ Department of Ophthalmology School of Medicine Flinders University Adelaide SA Australia; ^2^ Menzies Institute for Medical Research University of Tasmania Hobart Tas. Australia; ^3^ Department of Ophthalmology JDWNR Hospital Ministry of Health Thimphu Bhutan; ^4^ National Program for Eye Health Phnom Penh Cambodia; ^5^ Center for Sight Teaching Hospital Kandy Sri Lanka; ^6^ South Australian Institute for Ophthalmology University of Adelaide Adelaide SA Australia; ^7^ Sight For All Adelaide SA Australia; ^8^ Department of Haematology and Genetic Pathology School of Medicine Flinders University Adelaide SA Australia

**Keywords:** congenital cataract, inherited eye disease, mutation screening, next‐generation sequencing

## Abstract

**Background:**

Pediatric cataract is an important cause of blindness and visual impairment in children. A large proportion of pediatric cataracts are inherited, and many genes have been described for this heterogeneous Mendelian disease. Surveys of schools for the blind in Bhutan, Cambodia, and Sri Lanka have identified many children with this condition and we aimed to identify the genetic causes of inherited cataract in these populations.

**Methods:**

We screened, in parallel, 51 causative genes for inherited cataracts in 33 probands by Ampliseq enrichment and sequencing on an Ion Torrent PGM. Rare novel protein coding variants were assessed for segregation in family members, where possible, by Sanger sequencing.

**Results:**

We identified 24 rare (frequency <1% in public databases) or novel protein coding variants in 12 probands and confirmed segregation of variants with disease in the extended family where possible. Of these, six are predicted to be the cause of disease in the patient, with four other variants also highly likely to be pathogenic.

**Conclusion:**

This study found that 20%–30% of patients in these countries have a mutation in a known cataract causing gene, which is considerably lower than the 60%–70% reported in Caucasian cohorts. This suggests that additional cataract genes remain to be discovered in this cohort of Asian pediatric cataract patients.

## 1 INTRODUCTION

Pediatric cataract (including congenital cataract) is an opacity of the ocular lens that is present at birth (congenital) or develops during childhood. Prevalence estimates range from 0.33 to 22.7 per 10,000 (Sheeladevi, Lawrenson, Fielder, & Suttle, 2016) live births in populations across the world. It was previously thought that prevalence was around 10 times greater in low‐income economies than high‐income countries (Foster, Gilbert, & Rahi, 1997), but more recent reviews suggest that this is not the case with high prevalence reported in many higher income countries (Sheeladevi et al., 2016). Nonetheless, cataract is an important cause of childhood blindness globally and is one of the preventable and treatable conditions targeted by Vision 2020 programs (Gilbert & Foster, 2001).

Around 25% of pediatric cataract is inherited (Shiels & Hejtmancik, 2007) and over 100 genes have been reported for isolated pediatric cataract, with hundreds more for syndromic cataract (Shiels, Bennett, & Hejtmancik, 2010). Cataract‐causing genes include structural proteins of the crystalline lens as well as transport molecules, signaling proteins, and transcription factors. Analysis of panels of cataract‐causing genes in patients with inherited cataract detect mutations in 60%–70% of patients (Gillespie et al., 2014; Javadiyan et al., 2017; Ma et al., 2016), but these studies have been undertaken in cohorts of patients predominantly of European descent. Mutations in these same genes have been reported in patients from all over the world; however, large‐scale gene screening has not yet been undertaken in patients with pediatric cataract from developing countries.

Recently, surveys to document causes of childhood blindness have been undertaken in Bhutan (Farmer et al., 2015), Cambodia (Sia et al., 2010), and Sri Lanka (Gao et al., 2011). These studies identified many children in attendance at schools for the blind in these countries, with cataract as the underlying cause of their visual impairment. We investigated the genetic causes of cataract in children with suspected or known inherited cataract through screening of 51 genes known to cause this disease, using the same methodologies as applied to Australian pediatric cataract patients (Javadiyan et al., 2017).

## 2 MATERIALS AND METHODS

### 2.1 Ethical compliance

Consent for each participant was obtained from parent, guardian, or other authorized persons in the first language of the patient or parent. The study was approved in Australia by the Human Research Ethics Committees of the University of Adelaide and Flinders University in South Australia. In Bhutan, permission to visit schools was granted by the respective ministries of Health in Bhutan. Approval was also obtained from the Research Committees at the National Referral Hospital, Thimphu, Bhutan. In Cambodia, permission to visit schools was granted by the Ministry of Health, Cambodia and approval was obtained from the National Ethics Committee for Health Research in Cambodia. In Sri Lanka, permission to visit the schools was granted by each principal and ethics approval was obtained from the Faculty of Medicine, University Of Peradenya Ethical Review Committee. The study adhered to the tenets of the Declaration of Helsinki.

### 2.2 Participant selection

Children under 16 years of age attending blind schools in Bhutan (Farmer et al., 2015), Cambodia (Sia et al., 2010), and Sri Lanka (Gao et al., 2011) underwent an ocular examination and review of records as part of audits of the causes of childhood blindness in each community as described previously (Farmer et al., 2015; Gao et al., 2011; Sia et al., 2010). While all children were examined at the time of recruitment, it was not possible to access historical medical records to determine age of onset, or to interview the family for a detailed history. The recruitment of additional family members was only possible if the family decided to attend the school on the day of the survey.

Patients were included in this analysis if they were observed to have bilateral pediatric (or congenital) cataract, with or without other ocular or systemic features. Patients with aniridia were excluded, even if cataract was also present. Where possible, additional affected and unaffected family members were also examined and recruited. Saliva was collected from each participant in the DNA saliva collection kit (Oragene DNA saliva collection kit) and extracted using prepIT L2P (DNA Genotek Inc., Ottawa, ON, Canada).

### 2.3 Screening of cataract genes

Genes known to cause pediatric cataract in human or mouse were selected from the literature and the complete list is available in Table S1. Fifty‐one genes known to cause pediatric cataract were sequenced as described previously, (Javadiyan et al., 2017), using identical sequencing, variant filtering, and functional prediction methods to allow direct comparison between studies. PCR primers to amplify coding, 3′‐ and 5′‐ untranslated regions of the 51 genes were designed with the Ion AmpliSeq Designer tool v1.22 (Life Technologies, http://www.ampliseq.com) and used to prepare amplicon‐based sequencing libraries with the Ion AmpliSeq library kit version 2.0. Sequencing was performed on an Ion Torrent PGM using The Ion PGM Sequencing 200 Kit v2 and an Ion 318 chip (Life Technologies). The final assay design consisted of a total of 1,216 amplicons ranging from 125 to 225 bp, covering 94.3% of the 154.1 kb target sequence.

Sequence alignment, variant calling, and annotation were performed as described previously (Siggs et al., 2017). Briefly, reads were aligned to the human genome reference sequence 19 (hg19) and variants called using the Torrent Suite v3.6 tools and annotated with Ion Reporter v4.0.

Variants were prioritized for validation and further analyzed if they were predicted to be protein‐changing, and were absent or rare with minor allele frequency (MAF) <1% in dbSNP137 (https://www.ncbi.nlm.nih.gov/SNP/), the Exome Aggregation Consortium (ExAC) (http://exac.broadinstitute.org/), and gnomAD (http://gnomad.broadinstitute.org/). In addition, identified variants were compared with an in‐house list of common sequencing errors previously detected with this gene panel (Javadiyan et al., 2017).

### 2.4 Confirmation of variants

Sanger sequencing was used to confirm the presence of variants meeting the filtering criteria and to assess segregation of mutations in families. Forward and reverse primer sequences were designed using Primer3 (Koressaar & Remm, 2007; Untergasser et al., 2012). PCR and sequencing was conducted as described previously (Siggs et al., 2017). Sequence chromatograms of affected and unaffected individuals were compared to each other and the reference sequence (see Table S1 for GenBank accession numbers) using Sequencher v.5 (GeneCodes Corporation, Ann Arbor, MI, USA).

Two detected variants in *GALK1* in proband WW1 could not be assessed by Sanger sequencing due to difficulties with primer design for fragments suitable for capillary sequencing. These two variants were confirmed using Sequenom iPLEX GOLD chemistry on an Autoflex Mass Spectrometer at the Australian Genome Research Facility, Brisbane, Australia.

### 2.5 Functional predictions

Each confirmed segregating novel mutation was assessed for a potential functional effect on the predicted protein sequence using SIFT (Kumar, Henikoff, & Ng, 2009) (http://sift.jcvi.org/) and the HumDiv model of Polyphen‐2 (Adzhubei et al., 2010) (version 2.2.2) (http://genetics.bwh.harvard.edu.ezproxy.utas.edu.au/pph2/). The conservation of each altered amino acid was calculated using PhyloP as implemented in Mutation Taster (http://www.mutationtaster.org/) and available through the University of California Santa Cruz (UCSC) genome browser. PhyloP values between −14 and +6 indicate conservation at individual nucleotides, ignoring the effects of neighboring nucleotides. Amino acid conservation across species was visualized using the Mutation Taster website. Variants were also assessed against the recommendations of the American College of Medical Genetics and Genomics and the Association for Molecular Pathology (Richards et al., 2015).

All variants reported in this study have been submitted to the ClinVar database (https://www.ncbi.nlm.nih.gov/clinvar/; ClinVar accessions 3267773).

## 3 RESULTS

Thirty‐three probands with pediatric cataract were screened for mutations in the 51 reported cataract‐causing genes.

**Figure 1:**
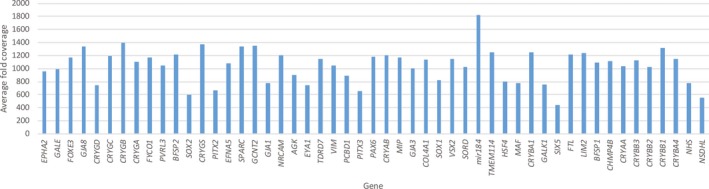
Average fold coverage of target genes sequenced from Ampliseq libraries in 33 Asian probands with pediatric cataract

The mean number of mapped reads per sample was 1,259,305 with 86% of reads on target. A mean of 892 reads was achieved per amplicon, with a coverage uniformity of 89%. Of all the amplicons, 93% and 88% were covered at least 20‐ and 100‐fold, respectively. The average coverage per gene is shown in Figure 1. Of the 1,216 amplicons, 27 amplicons (2%) across 15 genes were covered <20‐fold (Figure S1).

A total of 4,844 variants were annotated with an average of 146 variants per individual. In total, 188 variants were absent or rare (Minor Allele Frequency <1%) in publicly referenced databases including ExAC which contains 4,327 individuals from East Asian and 8,256 individuals from South Asian countries. Of the 188 variants, 90 were nonsynonymous exonic variants. Sixty‐five of these variants were removed due to their presence in our in‐house list of sequencing artifacts observed on the Ion Torrent platform with this gene panel. Of the remaining 25 variants, 24 variants were confirmed by Sanger sequencing in 12 probands (Table 1) and one was a false positive. The clinical phenotype observed in each proband is described in Table 2.

**Table 1:** Rare coding variants detected in probands with pediatric cataract

Country	Proband	Inheritance	Gene	Position in hg19	Nucleotide change	Protein change	Phylop	Seg	MAF in		Poly phen2	SIFT	ACMG
									ExAC	gnomAD			
Bhutan	P1	AD	*GJA8*	chr1:147380921	c.839C>G	p.(Pro280Arg)	5.36	Yes	0	0	ProbD	D	US
	E1	AD	*GALE*	chr1:24123216	c.766A>G	p.(Arg256Gly)	2.4	N/A	3.62 × 10^−5^	2.55 × 10^−5^	Benign	D	US
	WW1	AR	*GALK1*	chr17:73758836	c.742C>T	p.(Arg248Trp)	0.25	Yes	9.95 × 10^−5^	1.14 × 10^−4^	ProbD	D	US
			*GALK1*	chr17:73759221	c.485C>G	p.(Thr162Arg)	1.34	Yes	4.16 × 10^−5^	5.74 × 10^−5^	Benign	D	US
Cambodia	BB16cat		*GJA8*	chr1:147380102	c.20T>C	p.(Leu7Pro)	3.3	N/A	0	0	ProbD	D	LP
	PP50cat	AD	*MIP* a	chr12:56848301	c.97C>T	p.(Arg33Cys)	3.6	Yes	0	0	ProbD	D	LP
		AD	*COL4A1*	chr13:110864795	c.356C>G	p.(Pro119Arg)	5.27	No	0	0	ProbD	T	US
		AD	*NSDHL*	chrX:152037470	c.932T>C	p.(Val311Ala)	0.9	No	0	1.12 × 10^−5^	ProbD	T	US
	SR11cat		*PAX6*	chr11:31823289	c.177G>C	p.(Arg59Ser)	1.79	N/A	0	0	ProbD	T	US
	SR12cat	AD	*TDRD7*	chr9:100245251	c.2533C>G	p.(Gln845Glu)	3.5	No	1.65 × 10^−5^	4.07 × 10^−6^	ProbD	D	US
			*COL4A1*	chr13:110857844	c.900T>A	p.(Ser300Arg)	0.15	No	1.65 × 10^−5^	1.62 × 10^−5^	Benign	T	US
Sri Lanka	PCC10‐189	AR	*GCNT2* b	chr6:10626784	c.1153C>T	p.(Arg385Cys)	1.51	Yes	0	4.06 × 10^−6^	ProbD	D	US
	PCC01‐34		*HSF4*	chr16:67201678	c.910G>A	p.(Glu304Lys)	0.9	N/A	1.53 × 10^−4^	1.31 × 10^−4^	Benign	T	US
	PCC10‐188		*EPHA2* b	chr1:16464671	c.987_988insT	p.(Ser330Phefs*51)	–	N/A	0	8.32 × 10^−6^	–	–	–
			*GCNT2* b	chr6:10626784	c.1153C>T	p.(Arg385Cys)	1.51		0	4.06 × 10^−6^	ProbD	D	US
			*NHS*	chrX:17743727	c.1438C>T	p.(Arg480Cys)	5.33		1.82 × 10^−4^	1.75 × 10^−4^	ProbD	T	LB
	PCC01‐97A		*AGK*	chr7:141255292	c.26G>A	p.(Arg9Gln)	2.47	N/A	1.11 × 10^−3^	9.75 × 10^−4^	PossD	D	US
			*TDRD7*	chr9:100234592	c.1759G>T	p.(Asp587Tyr)	1.38		2.47 × 10^−5^	1.81 × 10^−5^	ProbD	D	US
			*PAX6*	chr11:31815036	c.982G>T	p.(Ala328Ser)	6.22		0	0	Benign	T	US
			*BFSP1*	chr20:17479645	c.776G>C	p.(Cys259Ser)	3.99		1.48 × 10^−4^	1.42 × 10^−4^	ProbD	D	US
			*CRYBB1*	chr22:27008146	c.186_188delGGT	p.(Val63del)	–		0	0	–	–	–
	PCC02‐105	AD	*CRYGD*	chr2:208986444	c.477_477delC	p.(Thr160Argfs*8)	0.75	Yes	0	0	–	–	–
			*CRYGD*	chr2:208986623	c.299G>A	p.(Gly100Asp)	4.96	No	0	0	ProbD	D	US
			*VIM*	chr10:17275680	c.719A>T	p.(Glu240Val)	5.2	No	1.65 × 10^−5^	8.12 × 10^−6^	ProbD	D	US

ACMG, American College Medical Genetics and Association for Molecular Pathology recommendations; B, Benign; D, deleterious; ExAC, Exome Aggregation Consortium; gnomAD, Genome Aggregation Database PolyPhen2 symbols; LB, likely benign; LP, likely pathogenic; N/A indicates no additional family members available for segregation or inheritance analysis; PossD, possibly damaging; ProbD, probably damaging; Seg, segregation in additional family; SIFT symbols; T, tolerated; US, uncertain significance.

a Mutation in MIP previously reported in a patient with congenital cataract.

b Variant is homozygous in the proband.

**Table 2:** Observed phenotypes and potentially pathogenic rare coding variants detected in pediatric cataract genes in probands

Country	Proband	Causative gene(s)	Phenotype	Age at diagnosis	Age at recruitment	Sex	Surgery	
							Right eye	Left eye
Bhutan	P1	*GJA8*	Congenital cataract with posterior capsule opacification	Birth	16	F	Yes	Yes
	E1	*Possibly GALE*	Congenital cataract, amblyopia, retinal dystrophy	Unknown	13	M	Yes	Yes
	WW1	*GALK1*	Congenital cataract of unknown etiology	Unknown	Not recorded	F	Yes	Yes
Cambodia	BB16cat	*GJA8*	Pediatric cataract	Unknown			–	–
	PP50cat	*MIP*	Pediatric cataract	Unknown		M	–	–
	SR11cat	*Possibly PAX6*	Pediatric cataract	Unknown			–	–
	SR12cat	*None segregating*	Pediatric cataract	Unknown		M	–	–
Sri Lanka	PCC10‐189	*Possibly GCNT2*	Congenital cataract with nystagmus	Birth	Not recorded	M	No	No
	PCC01‐34	*None predicted*	Pediatric cataract, no perception of light, minor phthisis, left corneal scar	Birth	11 years	M	–	–
	PCC10‐188	*EPHA2 or GCNT2*	Pediatric cataract	Birth	Not recorded	F	Yes	Yes
	PCC01‐97A	*Multiple; CRYBB1 most likely*	Pediatric cataract, microphthalmos and pseudophakia	Birth	6 years	F	Yes	Yes
	PCC02‐105	*CRYGD*	Bilateral congenital cataract	Unknown	7 years	F	No	No

### 3.1 Bhutanese probands

**Figure 2:**
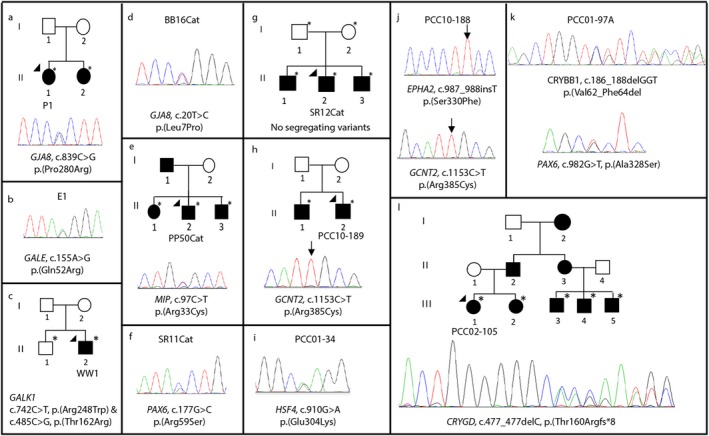
Segregating and likely causative variants in pediatric cataract patients and their families. The chromatograms show the variant detected by Sanger sequencing in the proband and any available family members. The gene names and mutation at cDNA and protein level are given. Solid circles indicate affected females and solid squares show the affected males. The arrowhead indicates the proband sequenced on the gene panel by Ampliseq. * indicates DNA available for segregation analysis. Homozygous variants are indicated on the chromatogram by an arrow. (a) *GJA8* variant in proband P1 and her sister; (b) *GALE* variant detected in patient E1; (c) compound heterozygous variants in *GALK1* in proband WW1 not present in unaffected brother. Variants confirmed by MassArray, no chromatogram available; (d) *GJA8* variant in BB16cat; (e) *MIP* variant segregates in family of proband PP50cat; (f) *PAX6* variant in SR11cat; (g) no segregating variants detected in the family of SR12cat; (h) Homozygous *GCNT2* variant in family of PCC10‐189; (i) *HSF4* variant in patient PCC01‐34 is likely benign; (j) Homozygous *EPHA2* and *GCNT2* variants in proband PCC10‐188; (k) Deletion of codon 63 of *CRYBB1* and novel missense mutation in *PAX6* in patient PCC01‐97A. Three other possible causative variants also detected; (l) *CRYGD* single base deletion segregates in the family of proband PCC02‐105

Of the five probands recruited, three had variants in the screened genes meeting the filtering criteria of rare and protein coding (Table 1). A novel mutation was detected in proband P1 in *GJA8,* c.839C>G, resulting in p.(Pro280Arg). The proband was described as having congenital cataract with posterior capsule opacification and has had surgery on both eyes (Table 2). The same mutation was detected in the proband's affected sister (Figure 2a) but the parents were not available for analysis. This variant is highly conserved and predicted to be damaging by both SIFT and Polyphen‐2 and is the most likely cause of disease in this family.

Variant c.766A>G resulting in p.(Arg256Gly) in *GALE* was detected in patient E1 (Figure 2b). Along with pediatric cataract, amblyopia and retinal dystrophy were reported in this patient indicating the syndromic nature of the disease. No other family members were available for assessment. The variant is well conserved and is predicted to be damaging by SIFT, but benign by Polyphen‐2. It has been reported in ExAC with a minor allele frequency of 0.000036 and 0.000025 in gnomAD. It cannot be said with certainty if this variant is responsible for the disease in this proband.

Two variants in *GALK1* were detected in proband WW1, c.742C>T coding for p.(Arg248Trp) and c.485C>G, coding for p.(Thr162Arg) (Figure 2c). Both of these variants were validated in the proband using MassArray genotyping due to technical infeasibility of Sanger sequencing. Both the variants are predicted to be deleterious by SIFT but only p.(Arg248Trp) is predicted to be damaging by PolyPhen‐2. However, these predictions do not take into account the combined presence of these very rare variants in the same patient with likely recessive inheritance. Neither variant was present in the proband's unaffected brother and together they likely account for autosomal recessive inheritance of cataract in this proband.

### 3.2 Cambodian probands

Fourteen probands from Cambodia were available for analysis and rare coding variants were observed in four patients.

Patient BB16cat had a novel variant in the well‐known cataract gene, *GJA8* (Figure 2d). Variant c.20T>C encoding p.(Leu7Pro) is well conserved and predicted to be pathogenic by multiple algorithms. No additional phenotypic information was available for this patient and family members were not available for genetic analysis, however, this mutation is highly likely to be the cause of the disease in this patient.

Patient PP50cat had a previously reported (Ma et al., 2016) mutation in *MIP* c.97C>T encoding p.(Arg33Cys). This well‐conserved variant is predicted to be pathogenic by both algorithms and segregates with cataract status in the pedigree (Figure 2e). Variants were also detected in *COL4A1* and *NSDHL* (Table 1), but did not segregate with cataract status in other family members, thus the *MIP* variant is the most likely cause of the disease in this family.

Proband SR11cat had a variant c.177G>C, p.(Arg59Ser) in *PAX6* (Figure 2f). The variant is novel, conserved, and is predicted to be pathogenic by PolyPhen‐2 but not SIFT. No detailed phenotypic information or additional family members were available for analysis. This variant may account for the disease, but requires further confirmation.

SR12cat had variants in two genes, *TDRD7* and *COL4A1*. Both variants are also reported in ExAC at low frequencies and neither segregated with the disease in the family (Figure 2g). These variants are unlikely to be the cause of the disease in this family.

### 3.3 Sri Lankan probands

Of the 14 available patients, rare coding variants of these genes were detected in five probands.

Proband PCC10‐189 was homozygous for the c.1153C>T variant coding for p.(Arg385Cys) in *GCNT2*. The proband's affected brother (individual II:1) was also homozygous for this novel variant (Figure 2h). No DNA was available from the parents, but neither was affected. This mutation likely represents the cause of the autosomal recessive cataract observed in these brothers.

The only variant detected in PCC01‐34 is a rare missense mutation in *HSF4* (Figure 2i). Although this gene is well known to cause cataract, given that the variant is present in the population, is in a poorly conserved region of the protein and is predicted to be tolerated by both algorithms, this variant is unlikely to be the cause of the disease in this patient. No additional family members were available for segregation analysis.

Variants in three genes were detected in proband PCC10‐188, a rare variant in *NHS* and novel variants in *EPHA2* and *GCNT2,* both in the homozygous state (Figure 2j). The cataract was present at birth in the proband and she had surgery in both eyes; however, no further details were available. Due to the nature of the mutation, the frameshift mutation in *EPHA2* c.987_988insT, p. (Ser330Phefs*51) is highly likely to be the cause of the disease, although this is difficult to confirm in the absence of additional family members. The mutation in *GCNT2* in PCC10‐188 is the same novel homozygous mutation that was found segregating in PCC‐189 and his brother (p.(Arg385Cys)). The possibility that this is a novel population specific variant should be considered; however, it is the most likely cause of disease detected in proband PCC‐189 and his brother.

Proband PCC01‐97A displays rare coding variants in five different cataract genes (Table 1). Three of the variants were rare in the population (in *AGK*,* TDRD7*, and *BFSP1*) and two (in *PAX6* and *CRYBB1)* were novel (Figure 2k). Cataract in this patient was present at birth and was described as syndromic (with microphthalmos and pseudophakia). The syndromic nature of the phenotype implicates *PAX6*, however, multiple other variants may be contributing to the phenotype in this patient with bioinformatic predictions suggesting that all mutations except the one in *PAX6* are likely to be functional. Given that the variant in *CRYBB1* is completely novel and results in the deletion of a whole codon, it may be considered the most likely causative variant. No other family members were available for the study, limiting the ability to interpret the findings of multiple variants.

Three variants were detected in two genes in PCC02‐105; two novel variants in *CRYGD* and one rare variant in *VIM* (Table 1). The frameshift mutation in *CRYGD,* c.477_477delC resulting in p.(Thr160Argfs*8) was the only variant present in the proband and his three affected cousins (Figure 2l) and as such is the probable cause of the disease in this family.

## 4 DISCUSSION

Thirty‐three probands with pediatric cataract were recruited during audits of the causes of childhood blindness in three countries—Bhutan, Cambodia, and Sri Lanka. Rare, coding variants were identified in 12 probands; however, these variants are likely to explain the cause of the disease in only six of these patients (P1 and WW1 from Bhutan; BB16cat and PP50cat from Cambodia; PCC02‐105 and PCC10‐188 from Sri Lanka) with a further four possibly solved (E1 from Bhutan; SR11 from Cambodia; and PCC01‐97A and PCC10‐189 from Sri Lanka). This equates to a rate of 18% (6/33) of cataract patients from the region having disease‐causing mutations in known cataract‐causing genes, and 30% if the possibly solved cases are included. This is substantially lower than the rates of 60%–70% reported in case series of European descent (Gillespie et al., 2014; Javadiyan et al., 2017; Ma et al., 2016). This difference remains evident even when compared with our previous study of Australian patients screened using the same gene panel, the same sequencing methodology, and the same variant filtering criteria, which found a success rate of 62% (Javadiyan et al., 2017). Gene discovery for this disease has predominantly been undertaken in patients of European descent and it is clear from this study that there remain novel genes to discover for inherited pediatric cataract in non‐European populations. We can hypothesize that such genes contribute to a higher proportion of disease in these relatively understudied populations. Families with multiple affected family members and clear phenotypes such as SR12 will be important in identifying such genes.

Alternatively, it is possible that a portion of the patients included in this study have nongenetic cataracts. The recruitment strategies employed made it difficult to obtain thorough family histories for all patients and in several cases, the inclusion of a child was made at the discretion of the examining clinician based on the information to hand. Up to 25% of cataract is inherited (Shiels & Hejtmancik, 2007) and the rate may be lower in areas without vaccination programs to prevent maternal rubella infection. This may mean that more children with this, or other nongenetic causes of cataract blindness, may have been inadvertently included in this study. Maternal rubella infection, however, does not account for the observations in this study. In 2012, Sri Lanka had a national rubella vaccination program, but Cambodia and Bhutan did not (Centres for Disease Control and Prevention, 2013). We found the highest rate of causative mutations in Bhutan (two of five probands). The audit also reported no cases of measles/rubella induced vision loss in the Bhutanese school children (Farmer et al., 2015). It remains to be determined how many of the probands with no detected mutations in this panel of genes have genetic forms of cataract and how many may be accounted for by environmental causes.

This study has thoroughly evaluated a large number of known cataract genes in a population which has not previously been studied to any extent, however, there are some limitations. The coverage of the target genes in this gene panel is high, but some regions could not be sequenced, either due to the inability to design amplicons in a given region, or the poor performance of some amplicons. A detailed assessment of the coverage and quality of sequencing using this panel on the Ion Torrent PGM has been published previously by our group (Javadiyan et al., 2017). Furthermore, the bioinformatic analysis can have a profound effect on the ability to detect variants. The algorithms for sequence alignment, variant calling, and variant annotation are constantly improving and these data should be reanalyzed in the future to detect variants missed in the current pipeline. The guidelines from the American College of Medical Genetics and Genomics and the Association for Molecular Pathology (Richards et al., 2015) classify most of the detected variants as variants of uncertain significance. This particular algorithm is designed to provide conservative findings in a clinical setting where results of genetic testing will be returned to patients and clinicians for use in the medical management of patients and genetic counseling of families. This necessarily requires caution when applying a label of pathogenic and only the highest levels of evidence are accepted. This tool reminds us to interpret the findings with caution and to seek additional evidence regarding the pathogenicity of these variants, but a classification of “uncertain significance” does not rule out a variant from being the true cause of disease.

This study has identified novel genetic mutations linked to pediatric cataract; p.(Pro280Arg) and p.(Leu7Pro) in *GJA8,* p.(Thr160Argfs*8) in *CRYGD*, p.(Arg385Cys) in *GCNT2*, p.(Ser330Phefs*51) in *EPHA2*, and very likely p.(Val62_Phe64del) in *CRYBB1*. This extends the known mutation spectrum for pediatric cataract. We also identified a recurrent mutation, p.(Arg33Cys) in *MIP*, segregating in a family from Cambodia. This mutation was previously reported de novo in a sporadic case of nonsyndromic bilateral congenital cataract (Ma et al., 2016) and in a multigeneration Chinese family with total congenital cataract (Gu et al., 2007).

Two novel variants were identified in the *CRYGD* gene in a family from Sri Lanka. The first, a frameshift mutation, p.(Thr160Argfs*8), clearly segregates with disease in the family and is the most likely cause of the disease in this family. The proband (PCC02‐105) also carries a second novel variant in this gene, which is also predicted to be pathogenic, p.(Gly100Asp). This second variant is not present in any of the other affected family members and thus is not likely to be the primary cause of the disease. This highlights the need to treat the interpretation of variants identified in only one individual with extreme caution. In terms of this study, this is applicable to patient BB16cat with a mutation in *GJA8* as well as the *EPHA2* variant in Sri Lankan proband PCC10‐188 and the novel variants in *PAX6* and *CRYBB1* in PCC01‐97A. The novel variants in *EPHA2* and *CRYBB1* are a frameshift and a deletion, respectively. Both genes and types of mutations have a strong tendency to be pathogenic for cataract; however, the *CRYBB1* mutation occurs in the context of other possibly pathogenic variants and additional evidence is required to determine if these novel variants are in fact the cause of disease.

We further identified three genes with recessive mutations in cataract patients. Proband WW1 from Bhutan is compound heterozygous for two mutations in *GALK1*. His unaffected brother does not carry either mutation. The parents were not available for testing; thus, it is not definitively determined if these variants are inherited in *cis* or *trans*; however, recessive mutations in this gene have been reported in many families with cataract previously, both as homozygous and compound heterozygous variants (Shiels et al., 2010). Both these variants are present at low rates in the ExAC database, consistent with a recessive inheritance pattern. The second recessive mutation identified in this cohort is in *GCNT2,* which is well known to cause recessive cataract (Shiels et al., 2010). The novel homozygous variant, p.(Arg385Cys), was detected in two brothers from Sri Lanka (PCC10‐189 and his brother), as well as a third affected child (PCC10‐188), not reported to be related but attending the same school for the blind. The presence of this homozygous novel variant in two “families” from the same school in Sri Lanka suggests that these two families are consanguineously related but this cannot be definitively determined from the data generated for this study. PCC10‐188 also carries a homozygous mutation in *EPHA2* (p.(Ser330Phefs*51)). This gene has been reported with this mode of inheritance in a family from Pakistan (Kaul et al., 2010). This homozygous variant was not present in ExAC at the time of generating the data; however, it has subsequently been identified in two individuals in the gnomAD resource. The homozygosity in proband PCC10‐188 further suggests the consanguinity of the parents of this child, in addition to the *GCNT2* variant. Either mutation is likely sufficient to cause cataract in this child.

There has been just one report of association between the deficiency of *GALE* and pediatric cataract in a 5.5‐year‐old girl with autosomal recessive pediatric cataract (Schulpis et al., 1993). Here, we report a missense variation, p.(Arg256Gly), in this gene in a proband from Bhutan with pediatric cataract, amblyopia, and retinal dystrophy (patient E1). Without additional family members, we cannot definitively determine a role for this variant in the disease; however, the variant has been reported at low frequency in public databases and is predicted by at least one algorithm to be benign. Coupled with the knowledge that this gene has only been reported previously in recessive disease, it seems unlikely that it is the sole cause of cataract and associated features in this proband.

We were able to determine the genetic cause in approximately 21% of pediatric cataract cases screened from three diverse Asian countries. It is probable that this mutation rate would be improved by more complete ascertainment of family members of affected probands. It is clear that while the known cataract genes do contribute to this disease in the Asian region and that novel variants exist in these populations, there is a clear need for further research to uncover the remaining genetic causes of the disease in this and other understudied regions.

## CONFLICTS OF INTEREST

The authors have no conflicts of interest to declare.
