# Supplementary material for: Identification of novel mutations causing pediatric cataract in Bhutan, Cambodia, and Sri Lanka
Source: Mol Genet Genomic Med. 2018 May 16;6(4):555–64. doi: 10.1002/mgg3.406 (PMC6081222; doi:10.1002/mgg3.406)
Supplement: Supplementary file 1 [file MGG3-6-555-s001.docx]

**Figure S1**: Amplicons (names are given by Ampliseq designer) with less than 20 fold coverage across 15 genes. The bars show the average amplicon coverage of 33 individuals screened.
