# Supplementary material for: Identification of novel mutations causing pediatric cataract in Bhutan, Cambodia, and Sri Lanka
Source: Mol Genet Genomic Med. 2018 May 16;6(4):555–64. doi: 10.1002/mgg3.406 (PMC6081222; doi:10.1002/mgg3.406)
Supplement: Supplementary file 2 [file MGG3-6-555-s002.docx]

**Table S1**: List of reported paediatric cataract genes selected for sequencing

| **Name** | **Genbank accession** | **OMIM accession** | **Locus** | **Reference** |
| --- | --- | --- | --- | --- |
| *GALE* | NM_000403.3 | 606953 | 1p36.11 | (Churchill and Graw, 2011) |
| *GALK1* | NM_016905.2 | 604313 | 17q25.1 | (Churchill and Graw, 2011)  (Stambolian, et al., 1995) |
| *NSDHL* | NM_015922.2 | 300275 | [Xq28](http://www.omim.org/geneMap/X/740?start=-3&limit=10&highlight=740) | (Churchill and Graw, 2011) |
| *PCBD1* | NM_000281.3 | 126090 | [10q22.1](http://www.omim.org/geneMap/10/233?start=-3&limit=10&highlight=233) | (Churchill and Graw, 2011) |
| *SORD* | NM_003104.5 | 182500 | [15q21.1](http://www.omim.org/geneMap/15/147?start=-3&limit=10&highlight=147) | (Churchill and Graw, 2011)  (Vaca, et al., 1982) |
| *CRYAA* | NM_013501.2 | 123580 | [21q22.3](http://www.omim.org/geneMap/21/117?start=-3&limit=10&highlight=117) | (Churchill and Graw, 2011)  (Litt, et al., 1998) |
| *CRYAB* | NM_009964.2 | 123590 | [11q23.1](http://www.omim.org/geneMap/11/755?start=-3&limit=10&highlight=755) | (Churchill and Graw, 2011)  (Berry, et al., 2001) |
| *CRYBA1* | NM_009965.2 | 123610 | [17q11.2](http://www.omim.org/geneMap/17/282?start=-3&limit=10&highlight=282) | (Churchill and Graw, 2011)  (Kannabiran, et al., 1998) |
| *CRYBB1* | NM_023695.2 | 600929 | [22q12.1](http://www.omim.org/geneMap/22/129?start=-3&limit=10&highlight=129) | (Churchill and Graw, 2011)  (Willoughby, et al., 2005) |
| *CRYBB2* | NM_007773.3 | 123620 | [22q11.23](http://www.omim.org/geneMap/22/113?start=-3&limit=10&highlight=113) | (Churchill and Graw, 2011)  (Litt, et al., 1997) |
| *CRYBB3* | NM_021352.3 | 123630 | [22q11.23](http://www.omim.org/geneMap/22/112?start=-3&limit=10&highlight=112) | (Churchill and Graw, 2011)  (Riazuddin, et al., 2005) |
| *CRYGA* | NM_014617.3 | 123660 | [2q34](http://www.omim.org/geneMap/2/745?start=-3&limit=10&highlight=745) | (Churchill and Graw, 2011) |
| *CRYGB* | NM_005210.3 | 123670 | [2q34](http://www.omim.org/geneMap/2/744?start=-3&limit=10&highlight=744) | (Churchill and Graw, 2011)  (AlFadhli, et al., 2012) |
| *CRYGC* | NM_007775.2 | 123680 | [2q33.3](http://www.omim.org/geneMap/2/739?start=-3&limit=10&highlight=739) | (Churchill and Graw, 2011) |
| *CRYGD* | NM_007776.2 | 123690 | [2q33.3](http://www.omim.org/geneMap/2/738?start=-3&limit=10&highlight=738) | (Churchill and Graw, 2011)  (Heon, et al., 1999) |
| *CRYGS* | NM_017541.2 | 123730 | [3q27.3](http://www.omim.org/geneMap/3/740?start=-3&limit=10&highlight=740) | (Churchill and Graw, 2011)  (Sun, et al., 2005) |
| *BFSP1* | NM_001195.3 | 603307 | [20p12.1](http://www.omim.org/geneMap/20/90?start=-3&limit=10&highlight=90) | (Churchill and Graw, 2011)  (Ramachandran, et al., 2007) |
| *BFSP2* | NM_003571.2 | 603212 | [3q22.1](http://www.omim.org/geneMap/3/542?start=-3&limit=10&highlight=542) | (Churchill and Graw, 2011)  (Jakobs, et al., 2000) |
| *COL4A1* | NM_001845.4 | 120130 | [13q34](http://www.omim.org/geneMap/13/259?start=-3&limit=10&highlight=259) | (Churchill and Graw, 2011) |
| *GJA1* | NM_000165.3 | 121014 | [6q22.31](http://www.omim.org/geneMap/6/696?start=-3&limit=10&highlight=696) | (Churchill and Graw, 2011) |
| *GJA3* | NM_021954.3 | 121015 | [13q12.11](http://www.omim.org/geneMap/13/13?start=-3&limit=10&highlight=13) | (Churchill and Graw, 2011)  (Mackay, et al., 1999) |
| *GJA8* | NM_005267.4 | 600897 | [1q21.2](http://www.omim.org/geneMap/1/811?start=-3&limit=10&highlight=811) | (Churchill and Graw, 2011)  (Shiels, et al., 1998) |
| *LIM2* | NM_005267.4 | 154045 | [19q13.41](http://www.omim.org/geneMap/19/806?start=-3&limit=10&highlight=806) | (Churchill and Graw, 2011)  (Pras, et al., 2002) |
| *MIP* | NM_012064.3 | 154050 | [12q13.3](http://www.omim.org/geneMap/12/435?start=-3&limit=10&highlight=435) | (Churchill and Graw, 2011)  (Berry, et al., 2000) |
| *NHS* | NM_001081052.1 | 300457 | [Xp22.13](http://www.omim.org/geneMap/X/94?start=-3&limit=10&highlight=94) | (Churchill and Graw, 2011)  (Burdon, et al., 2003) |
| *NRCAM* | NM_001193582.1 | 601581 | [7q31.1](http://www.omim.org/geneMap/7/480?start=-3&limit=10&highlight=480) | (Churchill and Graw, 2011) |
| *SPARC* | NM_003118.3 | 182120 | [5q33.1](http://www.omim.org/geneMap/5/555?start=-3&limit=10&highlight=555) | (Churchill and Graw, 2011) |
| *VIM* | NM_203472.1 | 193060 | [10p13](http://www.omim.org/geneMap/10/63?start=-3&limit=10&highlight=63) | (Churchill and Graw, 2011)  (Muller, et al., 2009) |
| *FOXE3* | NM_012186.2 | 601094 | [1p33](http://www.omim.org/geneMap/1/452?start=-3&limit=10&highlight=452) | (Churchill and Graw, 2011)  (Bremond-Gignac, et al., 2010) |
| *HSF4* | NM_012186.2 | 602438 | [16q22.1](http://www.omim.org/geneMap/16/432?start=-3&limit=10&highlight=432) | (Churchill and Graw, 2011)  (Bu, et al., 2002) |
| *MAF* | NM_005360.4. | 177075 | [16q23.2](http://www.omim.org/geneMap/16/528?start=-3&limit=10&highlight=528) | (Churchill and Graw, 2011)  (Jamieson, et al., 2002) |
| *PAX6* | NM_000280.4 | 607108 | [11p13](http://www.omim.org/geneMap/11/243?start=-3&limit=10&highlight=243) | (Churchill and Graw, 2011)  (Glaser, et al., 1994) |
| *PITX3* | NM_005029.3 | 602669 | [10q24.32](http://www.omim.org/geneMap/10/416?start=-3&limit=10&highlight=416) | (Churchill and Graw, 2011)  (Semina, et al., 1998) |
| *SIX5* | NM_175875.4 | 600963 | [19q13.32](http://www.omim.org/geneMap/19/651?start=-3&limit=10&highlight=651) | (Churchill and Graw, 2011) |
| *SOX1* | NM_005986.2 | 602148 | [13q34](http://www.omim.org/geneMap/13/264?start=-3&limit=10&highlight=264) | (Churchill and Graw, 2011) |
| *SOX2* | NM_003106.3 | 184429 | [3q26.33](http://www.omim.org/geneMap/3/700?start=-3&limit=10&highlight=700) | (Churchill and Graw, 2011) |
| *EPHA2* | NM_004431.3 | 176946 | [1p36.13](http://www.omim.org/geneMap/1/156?start=-3&limit=10&highlight=156) | (Churchill and Graw, 2011)  (Shiels, et al., 2008) |
| *EFNA5* | NM_001962.2 | 601535 | 5q21.3 | (Churchill and Graw, 2011) |
| *AGK* | NM_023538.2 | 610345 | [7q34](http://www.omim.org/geneMap/7/605?start=-3&limit=10&highlight=605) | (Aldahmesh, et al., 2012) |
| *GCNT2* | NM_145649 | 600429 | [6p24.3-p24.2](http://www.omim.org/geneMap/6/45?start=-3&limit=10&highlight=45) | (Pras, et al., 2004)  (Yu, et al., 2001) |
| *NECTIN3 (PVRL3)* | NM_001243288.1 | 607147 | [3q13.13](http://www.omim.org/geneMap/3/421?start=-3&limit=10&highlight=421) | (Churchill and Graw, 2011) |
| *EYA1* | NM_010164.2 | 601653 | [8q13.3](http://www.omim.org/geneMap/8/278?start=-3&limit=10&highlight=278) | (Azuma, et al., 2000) |
| *FTL* | NM_010240.2 | 134790 | [19q13.33](http://www.omim.org/geneMap/19/725?start=-3&limit=10&highlight=725) | (Nonnenmacher, et al., 2011) |
| *CHMP4B* | NM_176812.4 | 610897 | [20q11.22](http://www.omim.org/geneMap/20/171?start=-3&limit=10&highlight=171) | (Shiels, et al., 2007) |
| *FYCO1* | NM_024513.3 | 607182 | [3p21.31](http://www.omim.org/geneMap/3/194?start=-3&limit=10&highlight=194) | (Chen, et al., 2011) |
| *TMEM114* | NM_001146336.1 | 611579 | [16p13.2](http://www.omim.org/geneMap/16/142?start=-3&limit=10&highlight=142) | (Jamieson, et al., 2007) |
| *TDRD7* | NM_014290.2 | 611258 | [9q22.33](http://www.omim.org/geneMap/9/278?start=-3&limit=10&highlight=278) | (Lachke, et al., 2011) |
| *CRYBA4* | NM_021351.1 | 123631 | [22q12.1](http://www.omim.org/geneMap/22/130?start=-3&limit=10&highlight=130) | (Zhou, et al., 2010) |
| *VSX2* | NM_182894.2 | 142993 | [14q24.3](http://www.omim.org/geneMap/14/297?start=-3&limit=10&highlight=297) | (Ferda Percin, et al., 2000) |
| *PITX2* | NM_011098.3 | 601542 | [4q25](http://www.omim.org/geneMap/4/389?start=-3&limit=10&highlight=389) | (Reis, et al., 2012) |
| *MIR184* | NR_038997.1 | 613146 | 15q25.1 | (Hughes, et al., 2011) |
